# Association Between Irisin Level and Cognitive Function: A Systematic Review and Meta‐Analysis

**DOI:** 10.1002/brb3.70662

**Published:** 2025-07-22

**Authors:** Chengyan Han, Zining Zhou, Linlin Kong, Jing Lu, Xinyun Li

**Affiliations:** ^1^ School of Rehabilitation Hangzhou Medical College Hangzhou Zhejiang China; ^2^ Department of Neurology, The Second Affiliated Hospital Zhejiang University School of Medicine Hangzhou China; ^3^ Department of Psychiatry, The First Affiliated Hospital Zhejiang University School of Medicine Hangzhou China

**Keywords:** cognitive function, irisin, meta‐analysis, systematic review

## Abstract

**Background:**

Irisin is a newly discovered muscle factor, and more and more research has focused on the association between irisin and brain function. Our study aimed at investigating the correlation between irisin level and cognition.

**Methods:**

Five electronic databases were searched for the correlation between irisin level and cognition in humans. The primary outcome was the correlation between irisin level and global cognition. Secondary outcomes were the correlation between irisin level and each cognitive domain, the correlation between brain‐derived neurotrophic factor (BDNF) level and global cognition, and the correlation between irisin level and BDNF level. Correlation coefficient (*r*) was used as effect size with its 95% confidence intervals (CI).

**Results:**

There were a total of 11 articles that reported the correlation between irisin level and global cognition, and the pooled result showed a positive correlation among them (*r* = 0.26, 95% CI: 0.10–0.41). The subgroup analysis showed a positive correlation among non‐Asians (*r* = 0.28, 95% CI: 0.16–0.40), studies with small sample size (*r* = 0.35, 95% CI: 0.20–0.49), irisin measured from both serum (*r* = 0.32, 95% CI: 0.15–0.49) and cerebrospinal fluid (*r* = 0.27, 95% CI: 0.03–0.50), scales using Mini‐Mental State Examination (MMSE) (*r* = 0.36, 95% CI: 0.16–0.52), and study populations with diseases impairing cognition (*r* = 0.26, 95% CI: 0.07–0.43). Besides, our results indicated that there were no correlations between irisin level and cognitive domains, including immediate memory, short‐term memory, executive function, attention, language, and visual spatial ability (all *p* > 0.05). Additionally, the results suggested a positive correlation between BDNF level and global cognitive function (*r* = 0.27, 95% CI: 0.12–0.42), as well as the correlation between irisin level and BDNF level (*r* = 0.41, 95% CI: 0.24–0.56).

**Conclusions:**

This study implied that irisin has a positive effect on cognitive function, and the mechanism may be associated with promoting the expression of BDNF.

## Graphical abstract

Irisin is a newly discovered myokine, and the theory of muscle–brain interaction suggests that irisin may have an impact on the cognitive function. Our meta‐analysis showed that higher irisin levels in the blood and cerebrospinal fluid were associated with better cognitive function, and irisin may exert its effects by upregulating brain‐derived neurotrophic factor. In terms of cognitive subdomains, no significant correlations have been found between irisin levels and memory, executive function, attention, language, and visual spatial ability.

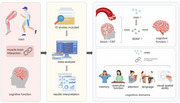

## 1 Introduction

Cognitive impairment can lead to abnormalities in various aspects such as memory, language, calculation, emotion, attention, orientation, and executive function, thereby having a negative impact on patients’ daily lives and social participation. In severe cases, it can be diagnosed as dementia, accompanied by behavior problems and personality changes. Many diseases will lead to cognitive impairment, such as neurodegenerative diseases, cerebrovascular diseases, traumatic brain injury, metabolic diseases, toxic injuries, and developmental abnormalities in children. Among them, Alzheimer's disease (AD) and vascular dementia (VD) are the two most common causes, especially in the elderly (Jia et al. 2020). Epidemiological data indicate that there are 55 million people worldwide suffering from dementia, and the prevalence rate is as high as 15% among people aged 65 and above (Buccellato et al. 2023; Palmer et al. 2022). At present, the main methods for the diagnosis of cognitive impairment are neuropsychological scales and imaging examinations. For example, Mini‐Mental State Examination (MMSE) and Montreal Cognitive Assessment (MoCA) are two of the most commonly utilized scales for examining global cognition, which refers to the overall mental capacity that encompasses multiple cognitive domains to process complex information, make judgments, and adapt to various cognitive tasks and environmental requirements. However, the current diagnostic methods are not sensitive to the early stages of cognitive impairment. In terms of treatment, although drug therapy and cognitive rehabilitation training are effective, it is currently incurable. Therefore, the research for a early diagnosis and treatment of cognitive impairment remains a hot topic.

Irisin was discovered in 2012 (Boström et al. 2012). It is a peptide and is regarded as a type of muscle factor (Waseem et al. 2022). Research has found that muscles secrete peroxisome proliferator‐activated receptor gamma coactivator 1 alpha (PGC‐1α) after exercise, which can regulate fibronectin type III domain containing protein 5 (FNDC5) to produce irisin (Liu et al. 2022). Early studies have made known that irisin is able to act on white adipocytes and induce their transformation into brown adipocytes, thus increasing thermogenesis (Kim et al. 2018). Currently, an increasing amount of research has focused on irisin, pointing out that irisin is also expressed in the heart, adipose tissue, brain, kidneys, and liver, involved in regulating blood sugar, fatty acid metabolism, cardiovascular function, and brain function (Ferrer‐Martínez et al. 2002; Xin et al. 2016). Studies have indicated that there is an interaction between muscles and the brain. For example, exercise can improve mood, sleep, memory, and cognition, with irisin being an important messenger in this process. Irisin, which is produced after exercise, not only strengthens the muscles and improves bone quality, but also enters the brain to act on certain regions such as the hippocampus (Maak et al. 2021). It is capable of regulating brain metabolic function, inhibiting neuroinflammation, reducing neuronal apoptosis, and enhancing synaptic plasticity through multiple signaling pathways (Qi et al. 2022). Thus, irisin plays a neuroprotective role in cerebral ischemic injury, neurodegenerative diseases, and neuropsychological disorders.

The research on the association between irisin and cognition was initially conducted in the context of AD. An article published in *Nature Medicine* has confirmed that irisin levels in both the hippocampus and cerebrospinal fluid are reduced in AD patients (Lourenco et al. 2019). Additionally, recent studies have demonstrated that irisin can also enhance cognition in patients with VD, frontotemporal dementia (FTD), and Parkinson's disease (PD) (Fraga et al. 2021; Guo et al. 2024). There are numerous hypotheses regarding how irisin improves cognition, one of which is through upregulating the expression of brain‐derived neurotrophic factor (BDNF) in the brain. These studies appear to offer new perspectives for the prediction and treatment of cognitive impairment.

On the basis of the aforementioned background, his systematic review and meta‐analysis was performed, in the hope of providing a relatively reliable conclusion on the correlation between irisin and cognitive function.

## 2 Methods

This study was conducted under the guidance of the Preferred Reporting Items for Systematic Reviews and Meta‐Analyses (PRISMA) statement and the Meta‐Analysis of Observational Studies in Epidemiology (MOOSE) guidelines. Besides, we have registered with PROSPERO in advance, and the registration number is CRD42024583826.

### 2.1 Search Strategy

We searched five databases, including PubMed, Embase, Web of Science, the Cochrane library, and Scopus. The search was from the inception of each database to August 17, 2024, with articles in all languages. “Irisin,” “FNDC5,” “fibronectin type III domain‐containing protein 5 precursor,” “cognition,” “cognitive,” “dementia,” “executive function,” “attention,” “memory,” and “information processing” were used as search terms. The search strategy is detailed in Table S1.

### 2.2 Selection Criteria

The selection criteria were as follows: (1) Research population: individuals with or without cognitive impairment; (2) Reporting the correlation coefficient between irisin levels and cognitive function; (3) The assessment of cognition could be global, or cognitive domains, like attention, executive function, memory, language, and visual spatial ability; (4) The assessment tools for cognitive function were objective and universal; (5) Source of samples: from plasma, serum, or cerebrospinal fluid, with enzyme‐linked immunosorbent assay (ELISA) as the method of measurement; (6) Research types: case–control, cohort, cross‐sectional studies and the baseline values of clinical trials, whereas reviews, case reports, and editorials were excluded; (7) Articles without full text or available data were excluded.

### 2.3 Outcome Indicators

The primary outcome was the correlation between irisin level and global cognitive function, with correlation coefficient (*r*) as the effect size.

The secondary outcomes were as follows: (1) The correlation between irisin level and cognitive domains, including memory, attention, executive function, language, and visual spatial ability; (2) The correlation between BDNF level and global cognition; (3) The correlation between irisin level and BDNF level.

### 2.4 Data Extraction

Two authors recorded data from each piece of literature independently, including first author, country, publication year, sample size, age, gender, complication, scale for assessment of cognition, and correlation coefficient between irisin level and cognition. Then, a third reviewer verified the data. If there was any dispute, the research team would discuss and make a decision. For example, different articles used different correlation coefficients, such as Pearson correlation coefficient and Spearman's rank correlation coefficient, and the two authors might have different methods of coefficient conversion. In such cases, the research team would discuss and adopt a unified standard for recording.

### 2.5 Quality Evaluation

According to the types of articles included in our study, different assessment tools were used to evaluate the risk bias. First, for case–control and cohort studies, Newcastle–Ottawa scale (NOS) was used. There are three parts (case–control studies: selection, comparability, exposure; cohort studies: selection, comparability, outcomes) with eight items in NOS scale and the total score is nine. It was considered high‐quality literature when the score was six or above. Second, the standards recommended by the Agency for Healthcare Research and Quality (AHRQ) were adopted for cross‐sectional studies. There are 11 items in AHRQ, with each result of “yes,” “no,” or “unclear.” If the result is “yes,” a score of 1 will be given. Studies with 8–11 scores were considered high quality. Finally, for clinical trials, the Cochrane Collaboration's risk of bias tool with six domains was used, including bias of selection, performance, detection, attrition, reporting, and others. The process above was also completed by two reviewers independently.

### 2.6 Statistical Analysis

Meta‐analysis was conducted with RevMan 5.3 and Stata 16.0. The pooled correlation coefficient (*r*) was calculated by inverse variance and Fisher's *Z* transformation, whereas the values of Fisher's *Z* were calculated from Pearson correlation coefficient, Spearman's rank correlation coefficient, or regression coefficient in each study. For different scales, we stated that the positive sign of correlation coefficient meant the higher the scores, the better the cognitive function. Heterogeneity assessment was conducted by Cochrane's *Q* test and *I* ^2^ value. It was considered high heterogeneity with *p* < 0.1 and *I* ^2^ > 50%. Subgroup analysis was performed by ethnicity, sample size, the sample source of irisin, scales, and study population. The sequential elimination method was adopted for sensitivity analysis. Publication bias was assessed by funnel plot and Egger's regression test. It was statistically significant when *p* < 0.05.

## 3 Results

### 3.1 Literature Search

**FIGURE 1:**
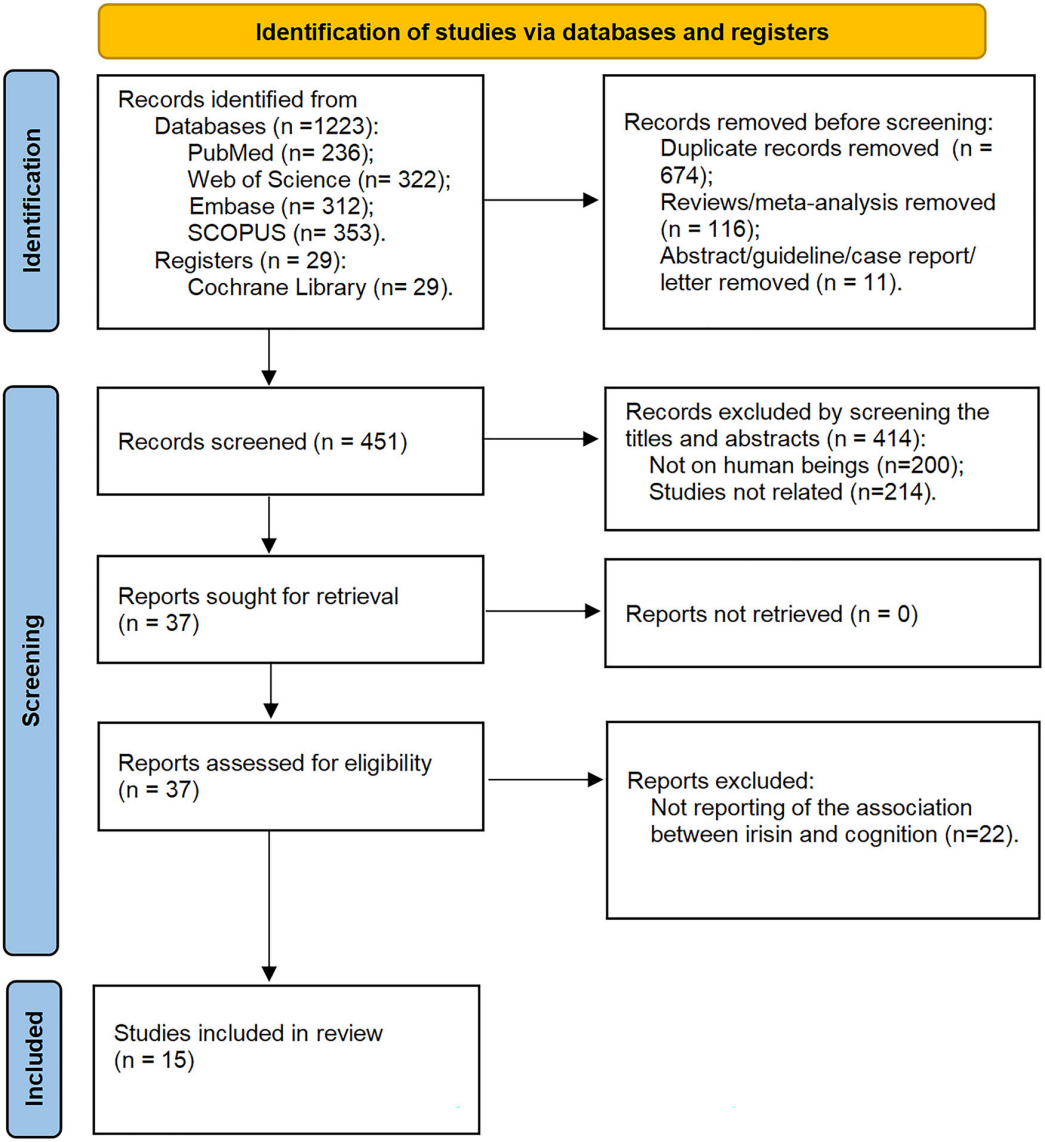
The process of literature screening.

A total of 1252 original articles were retrieved from PubMed (*n* = 236), Web of Science (*n* = 322), Embase (*n* = 312), Scopus (*n* = 353), and the Cochrane library (*n* = 29). Among them, 674 articles were duplicates and 127 articles did not match the selection criterion (6). Following the screening of the titles and abstracts, 414 articles were excluded, with 200 articles not meeting the selection criterion (1) and 214 articles not meeting the selection criterion (3), leaving 37 articles for full‐text reading. Furthermore, 22 articles were excluded according to selection criteria (2) and (7). Finally, 15 articles (Lin et al. 2019; Lourenco et al. 2020; Dicarlo et al. 2024; Belviranli et al. 2016; Fagundo et al. 2016; Faienza et al. 2021; Gonçalves et al. 2023; Ipekten et al. 2024; Kaloğlu et al. 2023; Küster et al. 2017; Lan et al. 2024; Li et al. 2024; Shi et al. 2024; Esad Tezcan et al. 2022; Zhang et al. 2021) were left, including 12 case–control studies, one cohort study, one cross‐sectional study, and one clinical trial (Figure 1).

### 3.2 Study Characteristics

Totally, 1304 samples from six countries were included in our meta‐analysis. The sample size of each study varied from 14 to 330 people. Eleven articles reported the correlation between irisin and global cognition, whereas eight articles pointed out the correlation between irisin and different cognitive domains. For the mechanism of the association between irisin and cognition, five articles showed the correlation between BDNF level and global cognitive function, and four articles reported the correlation between irisin level and BDNF level. For the source of irisin, five articles were from plasma, seven articles were from serum, and the remaining three articles were from cerebrospinal fluid. Seven articles used MMSE as the assessment tool of cognition, whereas four articles used MoCA, and two articles used other scales. The research subjects from four articles were healthy individuals, and the rest suffered from different diseases, such as AD, VD, and mild cognitive impairment (MCI). The details are summarized in Table 1.

**TABLE 1:** Study characteristics.

Study	Country	Study type	Sample size	Age	Male (%)	Study population	Assessment scales of cognition	Outcomes
Belviranli et al. (2016)	Turkey	Case–control	26	26.33 ± 4.08	100	Athletes and normal population	MMSE/IST	①⑤⑦⑧
Dicarlo et al. (2024)	Italy	Case–control	146	65.24 ± 9.48	41.78	People with or without AD	CDR‐SOB	①
Fagundo et al. (2016)	Spain	Case–control	49	29.04 ± 6.22	0	Obesity and healthy people	STROOP	③
Faienza et al. (2021)	Italy	Case–control	52	36.00 (20.00)	42.31	Adults with PWS	WAIS	①③⑤
Gonçalves et al. (2023)	Brazil	Case–control	36	71.2 ± 6.0	41.67	People with or without dementia	MMSE	①⑦⑧
Ipekten et al. (2024)	Turkey	Case–control	30	28.67 ± 1.50	/	Athletes and normal population	MMSE	①⑦⑧
Kaloğlu et al. (2023)	Turkey	Cross‐sectional	96	41.53 ± 10.85	66.67	Schizophrenia	VMPT/TMT‐A/TMT‐B	②③⑥
Küster et al. (2017)	Germany	Randomized controlled trial	47	71.2 ± 6.0	42.46	Normal elderly people	MMSE/ADAS‐CS/CVLT/WAIS/ECB	①②④⑦
Lan et al. (2024)	China	Cohort	330	8.29 ± 1.02	50.91	Healthy children	BRIEF	②③
Li et al. (2024)	China	Case–control	95	57.35 ± 9.84	60	T2DM with or without MCI	MMSE	①
Lin et al. (2019)	China	Case–control	133	58.84 ± 7.69	57.89	T2DM with or without MCI	MoCA/VFT/AVLT/TMT‐A/TMT‐B/DST/CDT	①②③④⑤⑥
Lourenco et al. (2020)	Brazil	Case–control	14	74.2 ± 7.1	71.43	AD	MMSE	①⑦⑧
Shi et al. (2024)	China	Case–control	100	60.80 ± 9.94	64.0	PD	MoCA	①
Esad Tezcan et al. (2022)	Turkey	Case–control	45	10.58 ± 1.90	64.44	ADHD	STROOP/SDLT/TDSM‐IV‐O	②③④
Zhang et al. (2021)	China	Case–control	105	62.0 ± 7.4	57.14	VD	MoCA	①

*Note*: ①, Correlation between irisin level and global cognition; ②, correlation between irisin level and memory; ③, correlation between irisin level and executive function; ④, correlation between irisin level and attention; ⑤, correlation between irisin level and verbal ability; ⑥, correlation between irisin level and visual spatial ability; ⑦, correlation between BDNF level and global cognition; ⑧, correlation between irisin level and BDNF level.

Abbreviations: AD, Alzheimer's disease; ADAS‐CS, Alzheimer's disease assessment scale‐cognitive subscale; ADHD, attention deficit hyperactivity disorder; AVLT, auditory verbal learning test; BRIEF, behavior rating inventory of executive function scale; CDR‐SOB, clinical dementia rating scale sum of boxes; CDT, clock drawing test; CVLT, California Verbal Learning Test; DST, digit span test; ECB, everyday cognition battery; FTD, frontotemporal dementia; IST, Isaacs’ Set Test of Verbal Fluency; MCI, mild cognitive impairment; MMSE, Mini Mental State Examination; MoCA, Montreal Cognitive Assessment; PD, Parkinson's disease; PWS, Prader‐Willi syndrome; SDLT, serial digit learning test; STROOP, Stroop Color Word Test; T2DM, type 2 diabetes mellitus; TDSM‐IV‐O, the Turgay DSM‐IV‐based screening and evaluation scale for attention deficit and disruptive behavior disorders‐parent form.

### 3.3 Methodological Quality Assessment

In the case–control studies, 12 articles were considered high quality with NOS scores ≥ 6, whereas another one was of medium quality with an NOS score of 5. The scores of these studies were mainly reduced due to the lack of representativeness and the failure to clarify whether the exposure measurements were blinded (Table 2). The cohort study included in our meta‐analysis was considered high quality with NOS score of 8 (Table S2). In addition, the cross‐sectional study had an AHRQ score of 6, which was considered medium quality (Table S3). The last one was a clinical trial, and the Cochrane Collaboration's risk of bias tool showed that the selection, detection, and attrition bias were low risk, whereas the performance bias was high risk (Table S4).

**TABLE 2:** Quality assessment of case–control studies with Newcastle–Ottawa scale (NOS) scores.

Item/Study		Belviranli et al. (2016)	Dicarlo et al. (2024)	Fagundo et al. (2016)	Faienza et al. (2021)	Gonçalves et al. (2023)	Ipekten et al. (2024)	Li et al. (2024)	Lin et al. (2019)	Lourenco et al. (2020)	Shi et al. (2024)	Esad Tezcan et al. (2022)	Zhang et al. (2021)
Selection	1. Adequate definition of cases	*	*	*	*	*	*	*	*	*	*	*	*
	2. Representativeness of cases					*		*	*		*		*
	3. Selection of controls	*	*	*		*	*				*	*	
	4. Definition of controls	*	*		*			*	*	*	*	*	*
Comparability	Control for important and additional factor	**	*	*	**	**	**	*	**	**	**	**	**
Exposure	1. Exposure assessment					*		*	*	*			
	2. Same method of ascertainment for cases and controls	*	*	*	*	*	*	*	*	*	*	*	*
	3. Non‐response rate	*	*	*	*	*	*	*	*	*	*	*	*
Total scores		7	6	5	6	8	6	7	8	7	8	7	7

*Note*: Each item can be awarded one star (*) when appropriately met. The Comparability item can receive a maximum of two stars (**). The detailed scoring criteria are shown in Table S6.

### 3.4 Correlation Between Irisin Level and Global Cognitive Function

**FIGURE 2:**
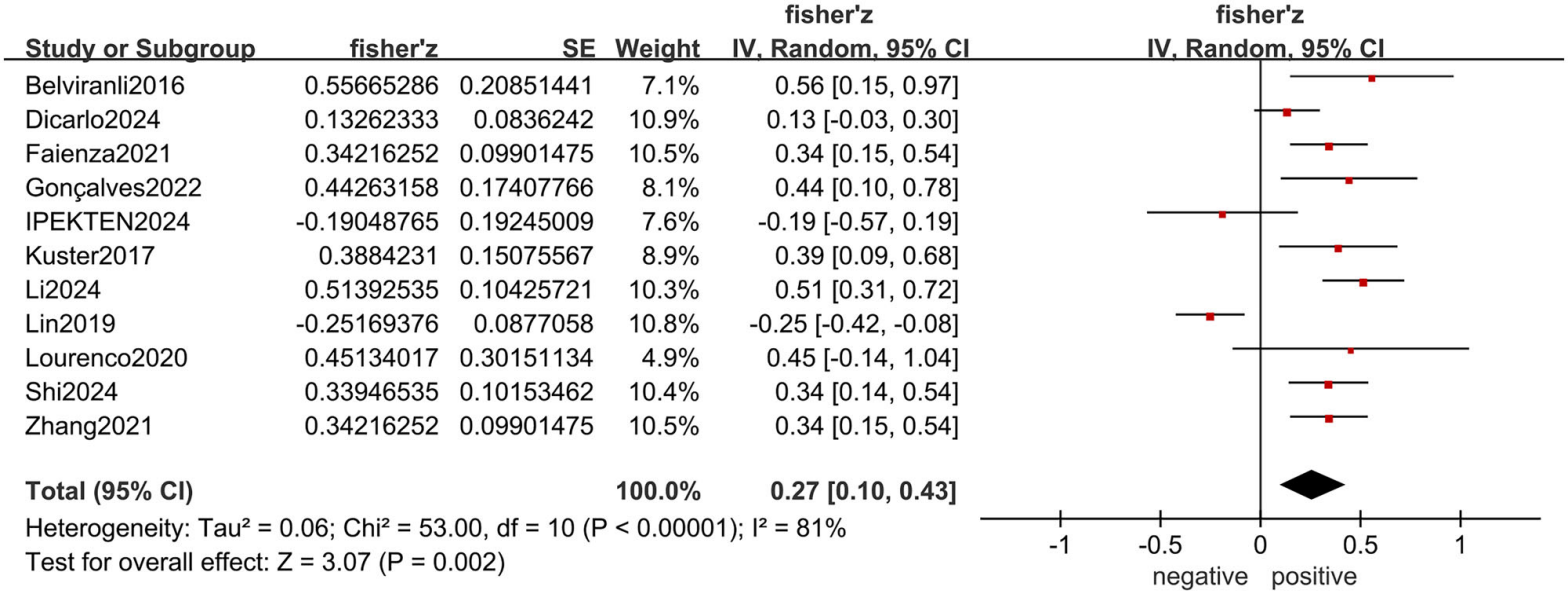
Forest plot of the correlation between irisin level and global cognitive function.

There were 11 articles (Lin et al. 2019; Lourenco et al. 2020; Dicarlo et al. 2024; Belviranli et al. 2016; Faienza et al. 2021; Gonçalves et al. 2023; Ipekten et al. 2024; Küster et al. 2017; Li et al. 2024; Shi et al. 2024; Zhang et al. 2021) reporting the correlation between irisin level and cognition, with seven (Belviranli et al. 2016; Faienza et al. 2021; Gonçalves et al. 2023; Küster et al. 2017; Li et al. 2024; Shi et al. 2024; Zhang et al. 2021) showing positive correlation, one (Lin et al. 2019) presenting negative correlation, and the remaining three (Lourenco et al. 2020; Dicarlo et al. 2024; Ipekten et al. 2024) showing no significant correlation. Because of the high heterogeneity (*I* ^2^ = 81%, *p* < 0.001), a random effects model was adopted. Meta‐analysis revealed that irisin level and global cognitive function were positively correlated (*r* = 0.26, 95% CI: 0.10–0.41, *p* = 0.002) (Figure 2).

The subgroup analysis showed a positive correlation between irisin level and global cognitive function among non‐Asians (*r* = 0.28, 95% CI: 0.16–0.40, *p* < 0.001), studies with small sample size (*r* = 0.35, 95% CI: 0.20–0.49, *p* < 0.001), irisin measured from both serum (*r* = 0.32, 95% CI: 0.15–0.49, *p* < 0.001) and cerebrospinal fluid (*r* = 0.27, 95% CI: 0.03–0.50, *p* = 0.03), scales with MMSE (*r* = 0.36, 95% CI: 0.16–0.52, *p* < 0.001), study populations with diseases impairing cognition (*r* = 0.26, 95% CI: 0.07–0.43, *p* = 0.007), whereas there was no correlation among Asians, studies with large sample size, irisin measured from plasma, scales with others, and study populations without disease impairing cognition (all *p* > 0.05) (Table 3).

**TABLE 3:** Subgroup analysis of the correlation between irisin level and global cognitive function.

Subgroup	No. of studies	Heterogeneity		Meta‐analysis		Heterogeneity between subgroups	
		*I* ^2^	*p* value	*r*(95% CI)	*p* value	*I* ^2^	*p* value
Ethnicity							
Asians (Lin et al. 2019, Belviranli et al. 2016, Ipekten et al. 2024, Li et al. 2024, Shi et al. 2024, Zhang et al. 2021)	6	89%	<0.001	0.22(−0.07 to −0.46)	0.14	0%	0.65
Non‐Asians (Lourenco et al. 2020, Dicarlo et al. 2024, Faienza et al. 2021, Gonçalves et al. 2023, Küster et al. 2017)	5	23%	0.27	0.28(0.16–0.40)	<0.001		
Sample size							
≥100 (Lin et al. 2019, Dicarlo et al. 2024, Shi et al. 2024, Zhang et al. 2021)	4	89%	<0.001	0.14(−0.14 to −0.39)	0.33	49.2%	0.16
<100 (Lourenco et al. 2020, Belviranli et al. 2016, Faienza et al. 2021, Gonçalves et al. 2023, Ipekten et al. 2024, Küster et al. 2017, Li et al. 2024)	7	48%	0.07	0.35(0.20–0.49)	<0.001		
The sample source of irisin							
Plasma (Lin et al. 2019, Belviranli et al. 2016, Shi et al. 2024)	3	92%	<0.001	0.19(−0.30 to −0.68)	0.44	0%	0.86
Serum (Faienza et al. 2021, Ipekten et al. 2024, Küster et al. 2017, Li et al. 2024, Zhang et al. 2021)	5	62%	0.03	0.32(0.15–0.49)	<0.001		
Cerebrospinal fluid (Lourenco et al. 2020, Dicarlo et al. 2024, Gonçalves et al. 2023)	3	39%	0.19	0.27(0.03–0.50)	0.03		
Scales							
MMSE (Lourenco et al. 2020, Belviranli et al. 2016, Gonçalves et al. 2023, Ipekten et al. 2024, Küster et al. 2017, Li et al. 2024)	6	56%	0.05	0.36(0.16–0.52)	<0.001	29.4%	0.23
Others (Lin et al. 2019, Dicarlo et al. 2024, Faienza et al. 2021, Shi et al. 2024, Zhang et al. 2021)	5	88%	<0.001	0.18(−0.05 to −0.39)	0.13		
Study populations							
With disease impairing cognition (Lin et al. 2019, Lourenco et al. 2020, Dicarlo et al. 2024, Faienza et al. 2021, Gonçalves et al. 2023, Li et al. 2024, Shi et al. 2024, Zhang et al. 2021)	8	84%	<0.001	0.26(0.07–0.43)	0.007	0%	0.94
Without disease impairing cognition (Belviranli et al. 2016, Ipekten et al. 2024, Küster et al. 2017)	3	76%	0.02	0.24(−0.17 to −0.58)	0.24		

### 3.5 Correlation Between Irisin Level and Cognitive Domains

**FIGURE 3:**
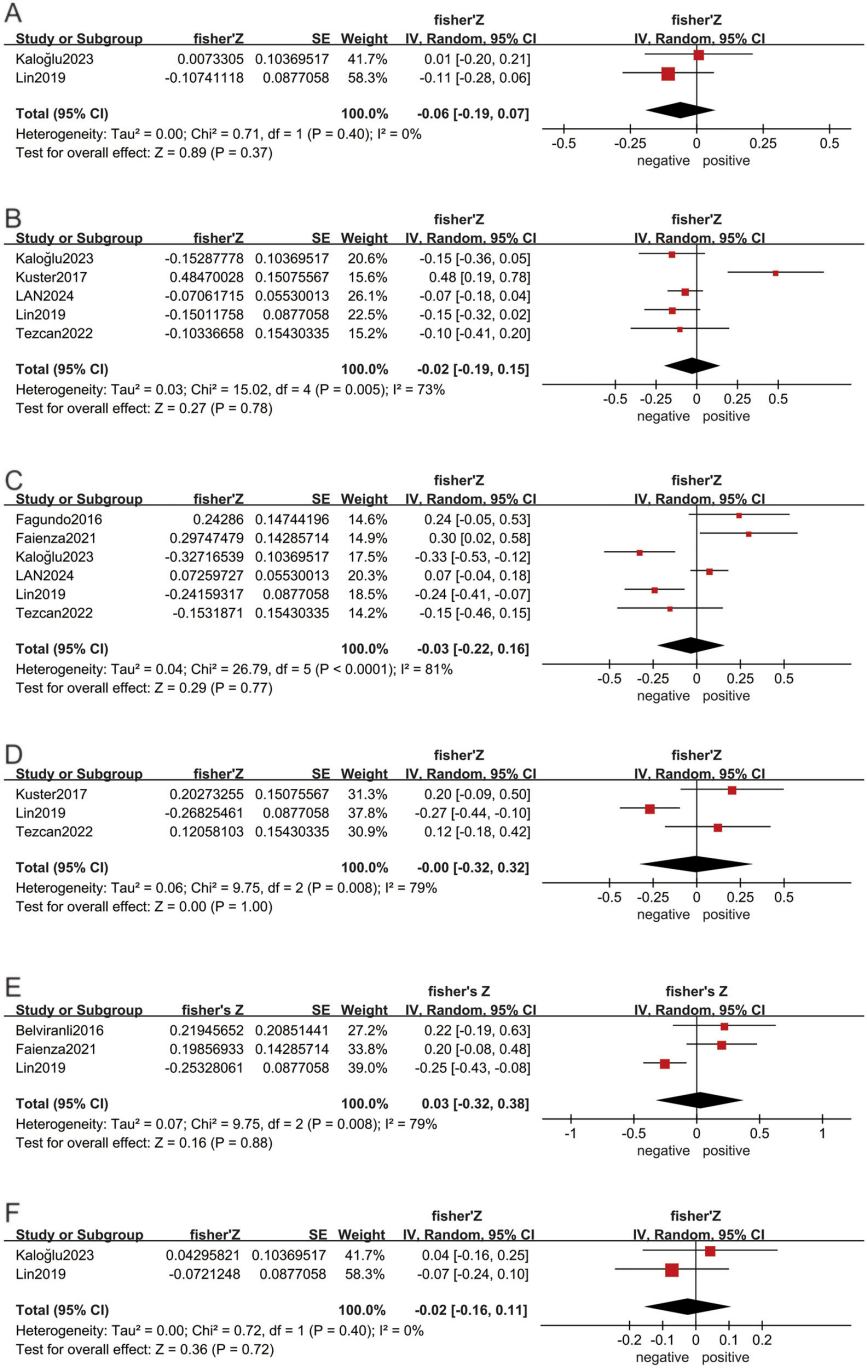
Forest plots of the correlation between irisin level and cognitive domains. Correlations between irisin level and immediate memory (A), short‐term memory (B), executive function (C), attention (D), language (E), and visual spatial ability (F).

There were eight articles (Lin et al. 2019; Belviranli et al. 2016; Fagundo et al. 2016; Faienza et al. 2021; Kaloğlu et al. 2023; Küster et al. 2017; Lan et al. 2024; Esad Tezcan et al. 2022) reporting the correlation between irisin and different cognitive domains. The pooled results showed that there was no significant correlation between irisin level and immediate memory, short‐term memory, executive function, attention, language, and visual spatial ability (all *p* > 0.05) (Figure 3).

### 3.6 Correlation Between BDNF Level and Global Cognitive Function

**FIGURE 4:**
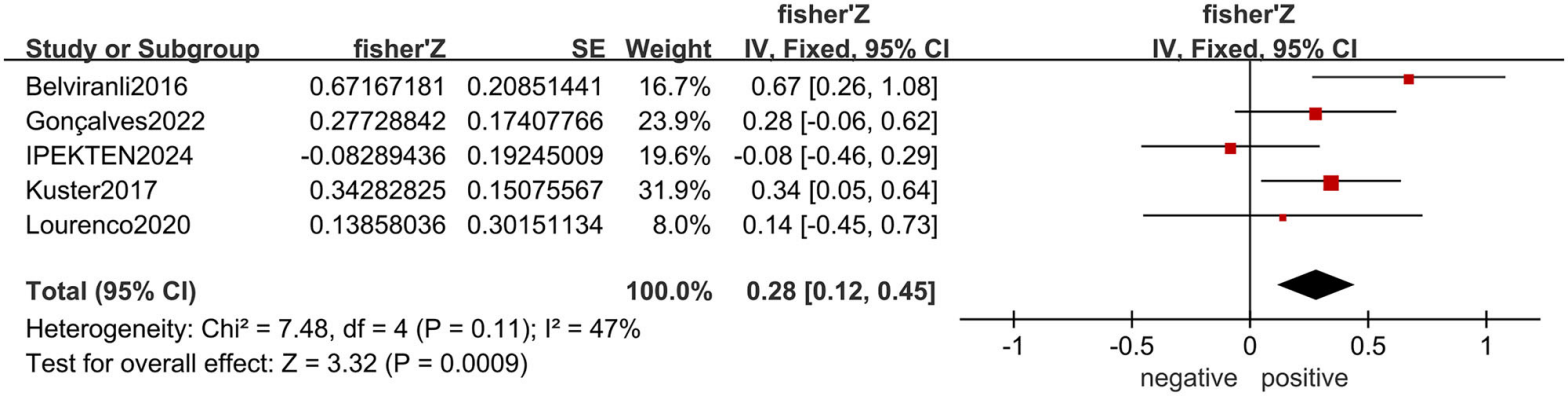
Forest plot of the correlation between BDNF level and global cognitive function.

Five articles (Lourenco et al. 2020; Belviranli et al. 2016; Gonçalves et al. 2023; Ipekten et al. 2024; Küster et al. 2017) showed the correlation between BDNF level and global cognition. A fixed effects model was used for the low heterogeneity (*I* ^2^ = 47%, *p* = 0.11), and the results indicated a positive correlation between BDNF level and global cognition (*r* = 0.27, 95% CI: 0.12–0.42, *p* < 0.001) (Figure 4).

### 3.7 Correlation Between BDNF Level and Irisin Level

**FIGURE 5:**
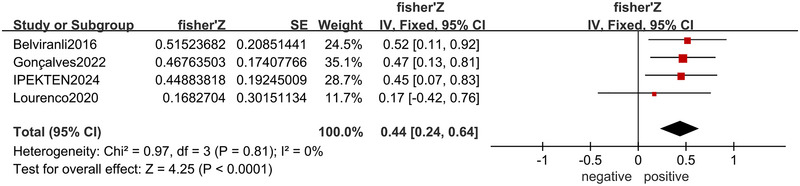
Forest plot of the correlation between BDNF level and irisin level.

There were four articles (Lourenco et al. 2020; Belviranli et al. 2016; Gonçalves et al. 2023; Ipekten et al. 2024) reporting the correlation between BDNF level and irisin level. The results showed that BDNF level and irisin level were positively correlated (*r* = 0.41, 95% CI: 0.24–0.56, *p* < 0.001), with a fixed effects model (*I* ^2^ = 0%, *p* = 0.81) (Figure 5).

### 3.8 Sensitivity Analysis

When the included studies were omitted one by one, there was no obvious change in the pooled results of the primary outcome. What's more, the heterogeneity was significantly decreased when excluding the study of Lin et al. (2019), indicating that this study might be the source of heterogeneity (Table S5).

### 3.9 Publication Bias

**FIGURE 6:**
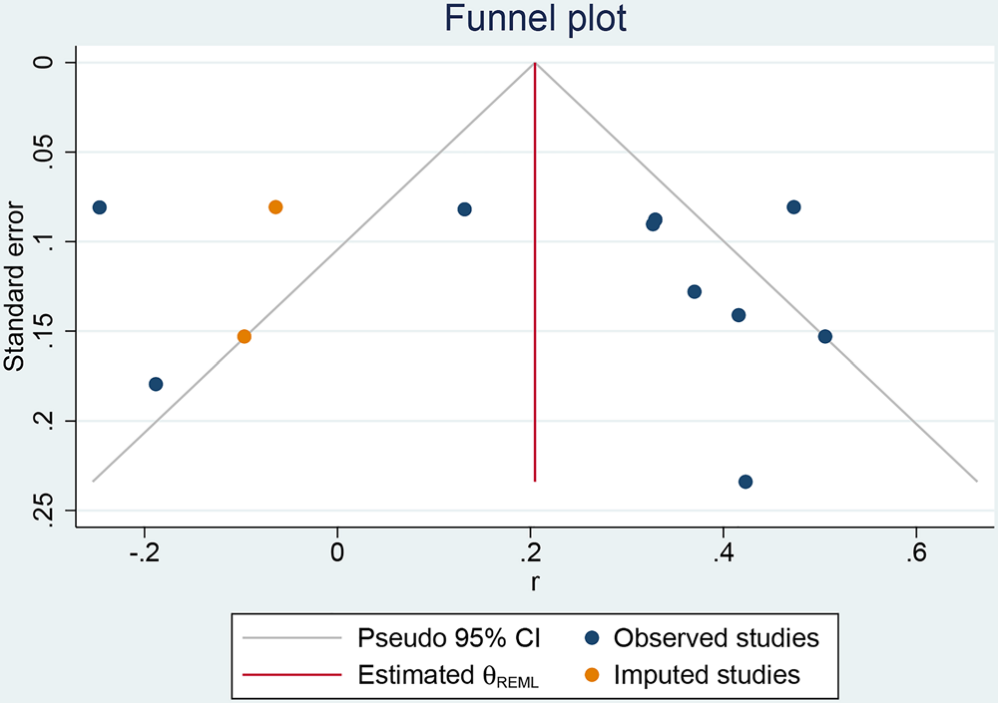
Funnel plot with trim and fill method of the correlation between irisin level and global cognitive function.

Egger's test showed that there was no significant publication bias (*z* =  0.34, *p* > 0.05). Trim and fill method indicated that after adding two virtual studies, the association between irisin level and global cognitive function was still positively correlated, which was similar to the original result, proving that the result was stable (Figure 6).

## 4 Discussion

In recent years, research on the association between irisin and brain function has been gradually increasing. However, there has been no previous meta‐analysis on the correlation between irisin level and cognition; thus, our study is the first one to focus on that, which will provide reference for future research in this field. Our research suggested that irisin level was positively correlated with global cognitive function, and the mechanism might be related to promoting the expression of BDNF. Furthermore, no statistical correlation was found between irisin level and each cognitive domain.

Irisin is secreted by many organs in human body, such as skeletal muscles, liver, and some regions of the brain, including hippocampus, which is meaningful for cognition. Many articles have proved the beneficial effects of exercise on neurodegenerative diseases, like AD and PD (Ahlskog 2011; Buchman et al. 2012). Current research has found that exercise can significantly promote the expression of irisin in skeletal muscles (Wrann et al. 2013). Adilakshmi et al. (2023) conducted a study comparing high intensity resistance training (HIRT) with endurance exercise in healthy individuals for 8 weeks and found that the serum irisin level in the HIRT group increased significantly. Another study has shown that the combination of aerobic exercise, endurance exercise, and balance exercise can maintain the irisin level in the serum of diabetic patients, with the improvement of MOCA scores (Ghodrati et al. 2023). Besides, it has been proved that Tai chi exercise for 10 weeks can increase irisin and BDNF level in the serum of the elderly and improve their attention (Solianik et al. 2022). These studies have confirmed the positive effect of irisin induced by exercise on cognitive function, supporting the findings of our research.

Previous meta‐analyses have made conclusions that ethnicity is one of the sources of heterogeneity (Hu et al. 2020; Cui et al. 2024). Similarly, our subgroup analysis suggested that irisin level was positively correlated with global cognitive function in non‐Asians, whereas there was no statistical correlation in Asians. However, there has been no research to explain the differences caused by ethnicity.

In subgroup analysis conducted by sample size, studies with small sample size showed a positive correlation between irisin level and global cognitive function, whereas studies with large sample size did not show correlation, suggesting that more studies were needed to obtain more reliable conclusions.

Additionally, the results of subgroup analysis indicated that whether irisin was measured from the serum or cerebrospinal fluid, it was positively correlated with global cognition, whereas there was no correlation when irisin was measured from plasma. At present, there are no relevant studies to confirm the difference in the irisin level in serum and plasma. During the process of blood coagulation, various cellular components and coagulation factors participate in the reaction, which may affect the release or binding state of irisin, thus leading to differences in the irisin level between them.

There are a considerable number of scales to assess the cognitive function, among which MMSE is the most widely used one (Creavin et al. 2016). Our subgroup analysis demonstrated that studies using MMSE instead of other scales had a positive correlation between irisin level and global cognition. In the subgroup of non‐MMSE, the heterogeneity might increase due to the different reliability and validity of the different scales, which would have negative influence on the results.

In our meta‐analysis, both healthy and diseased groups were included as study population. Subgroup analysis showed a positive correlation in people suffering from diseases which could impair the cognition, whereas there was no correlation in healthy individuals. The reason might be that the differences of cognitive function among the healthy were not significant enough, so that the sensitivity of the scales was limited in healthy people.

Different regions of the brain are related to different cognitive domains for example, the hippocampus plays a role in memory, the prefrontal cortex is associated with executive function, and the posterior parietal lobe and brainstem are linked to attention (Harvey 2019). Some of the included articles calculated the correlation coefficient between irisin and cognitive domains. Therefore, we attempted to conduct meta‐analysis in memory, execution, language ability, visual spatial ability, and attention, in order to draw more accurate conclusions. However, our results showed no correlation in each cognitive domain. Due to the small study size, the results might be limited.

Previous articles have explored the mechanism by which irisin improves cognitive function. Irisin in the peripheral circulation is able to cross the blood–brain barrier and enter the central nervous system to act on the brain (Islam et al. 2021). First, studies have shown that irisin is associated with neurogenesis. It is believed that irisin is a potential effector of hippocampal neurogenesis by upregulating the expression of neurogenic markers, such as cFos, Arc, and Zif268 (Poo et al. 2016). In vitro, it has been found that irisin can promote the proliferation of hippocampal neurons by regulating some signaling pathways (Moon et al. 2013). Besides, irisin is able to inhibit neuroinflammation. Microglia and astrocytes play a leading role in the process of neuroinflammation, which will release inflammatory factors such as interleukin‐1β (IL‐1β), tumor necrosis factor‐α (TNF‐α), and nitric oxide (NO) when they are activated. On the other hand, IL‐10 and IL‐6 are common anti‐inflammatory factors that can inhibit the activation of microglia. Research has shown that irisin has the effect of alleviating neuroinflammation by upregulating the expression of IL‐10 and IL‐6 (Pignataro et al. 2021). Moreover, autophagy is a self‐protective strategy of cells and it was proved that autophagy could reduce amyloid protein accumulation and improve cognitive abilities in mouse models of AD (Rocchi et al. 2017). Irisin is able to upregulate the expression of optic atrophy 1 (Opa1), thus inducing mitophagy (Xin and Lu 2020). Irisin also exerts an anti‐inflammatory effect through BDNF (Wrann et al. 2013). In addition, a study has indicated that after a month of treatment with recombinant irisin, the behavioral performance of mice in the tail suspension test, forced swimming test, and open field test was improved, indicating the beneficial effect of irisin on the neuropsychiatric diseases, which might be related to the upregulation of the levels of endogenous brain factors and anti‐inflammatory cytokines (Pignataro et al. 2022).

One of the most widely recognized mechanisms is that irisin improves cognition by upregulating the expression of neurotrophic factors, which can promote neurogenesis, regulate synaptic plasticity, and alleviate neuroinflammation (Wei et al. 2024). BDNF, a common neurotrophic factor, is meaningful for maintaining neuronal homeostasis and is associated with cognitive function (Michalski and Fahnestock 2003). The BDNF gene is translated into pro‐BDNF, which is then cleaved into mature BDNF (mBDNF). Pro‐BDNF promotes apoptosis and inhibits neurite growth by binding to the p75 NTR receptor, whereas mBDNF facilitates synaptogenesis and neurite growth (Gao et al. 2022). Animal experiments have shown that the level of mBDNF may be 10 times higher than that of pro‐BDNF (Matsumoto et al. 2008; Dieni et al. 2012). Research has made known that mBDNF can act on microglia to show anti‐inflammatory effects through multiple inflammatory signaling pathways (Lima Giacobbo et al. 2019). First, mBDNF binds to tropomyosin receptor kinase B (TrkB), inducing the activation of extracellular signal‐regulated kinase (ERK) and the phosphorylation of cAMP response element‐binding protein (CREB). Subsequently, it inhibits the activity of nuclear factor‐kappa B (NF‐κB) and the transcription of anti‐inflammatory genes. Besides, mBDNF may exert its anti‐inflammatory effect through the protein kinase B (Akt) signaling pathway and by blocking the activity of glycogen synthase kinase 3 (GSK‐3). It was found that the levels of both pro‐BDNF and mBDNF in the parietal cortex of patients with AD and MCI decreased, and the levels of these two were positively correlated with the cognitive function of the patients (Peng et al. 2005). Furthermore, the injection of recombinant mBDNF into HD animal models could improve the neurobehavioral performance (Giampà et al. 2013). Another study pointed out that the secretion of mBDNF increased during exercise, and it could protect neurons and glial cells from brain injury (Jo and Song 2021). Additionally, research has proved that irisin can promote the expression of BDNF in the brain. Natalichio et al. (2020) found a significant increase of BDNF mRNA in the brain by injecting irisin (0.5 µg/g) intraperitoneally into mice for 14 days. Dicarlo et al. (2023) also found that after short‐term subcutaneous administration of irisin to healthy mice subjected to stressful situations, the BDNF mRNA level in the prefrontal cortex increased significantly, and the mice's depression‐like behaviors were mitigated.

Our meta‐analysis showed that BDNF level was positively correlated with global cognitive function, whereas it was also positively correlated with irisin level, demonstrating that irisin may improve cognition by upregulating BDNF. However, after our verification of the original studies, we found that most of them measured total BDNF, whereas the remaining few cases were difficult to confirm whether they were mBDNF or total BDNF. Although the majority of total BDNF is mBDNF, with the opposite physiological effects of pro‐BDNF and mBDNF, the analysis of the results was confusing.

This study had several advantages. First of all, the outcomes of our study were quite multiple. We not only analyzed the correlation between irisin level and global cognition but also conducted meta‐analysis in different cognitive domains. Furthermore, we verified the hypothesis that irisin improves cognition through the signaling pathway of BDNF. In addition, the quality assessments of the literature included in our study were mostly of high quality, and the results of sensitivity test were robust, indicating that our results were relatively reliable.

However, there were some shortcomings in our meta‐analysis. With the limited number of original research, especially in the analysis of each cognitive domain, the credibility of the conclusion might be influenced. Similarly, in order to ensure sufficient study size, we had no restrictions on the health status of the study population. However, some diseases may have a certain impact on the cognitive function. Therefore, meta‐analysis with study population of single disease only is necessary. Besides, as we could not fully verify the types of BDNF in all the included literature, we were unable to separately determine the impacts of pro‐BDNF and mBDNF on cognitive function, as well as their correlations with irisin. Finally, apart from the irisin/BDNF pathway, we have not verified other hypotheses regarding the mechanism, which need to be further investigated.

## 5 Conclusions

This study confirms that irisin has a positive effect on cognitive function, and it may be related to upregulating the expression of BDNF. It provides a new direction for the biological molecular diagnosis and drug discovery of cognitive impairment. We hope for more research in different cognitive domains and more focus on the mechanism of the association between irisin and cognition in the future.

## Author Contributions

**Chengyan Han**: conceptualization, software, formal analysis, writing – original draft preparation, funding acquisition. **Zining Zhou**: methodology, software, data curation. **Linlin Kong**: validation, writing – original draft preparation. **Jing Lu**: validation, writing – review and editing, supervision, funding acquisition. **Xinyun Li**: conceptualization, writing – review and editing, project administration. All authors have read and agreed to the published version of the manuscript.

## Peer Review

The peer review history for this article is available at https://publons.com/publon/10.1002/brb3.70662

## Supporting information




**Supporting Table 1**: Detailed search strategy
**Supporting Table 2**: Quality assessment of cohort study with NOS scores
**Supporting Table 3**: Quality assessment of cross‐sectional study with AHRQ score
**Supporting Table 4**: Quality assessment of randomized controlled trial with the Cochrane Collaboration's risk of bias tool
**Supporting Table 5**:Sensitivity analysis of the correlation between irisin level and global cognition

## Data Availability

The data that support the findings of this study are available from the corresponding author upon reasonable request.
