# Supplementary material for: Association Between Irisin Level and Cognitive Function: A Systematic Review and Meta‐Analysis
Source: Brain Behav. 2025 Jul 22;15(7):e70662. doi: 10.1002/brb3.70662 (PMC12284321; doi:10.1002/brb3.70662)
Supplement: Supplementary file 1 — Supporting Table 1: Detailed search strategy Supporting Table 2: Quality assessment of cohort study with NOS scores Supporting Table 3: Quality assessment of cross‐sectional study with AHRQ score Supporting Table 4: Quality assessment of randomized controlled trial with the Cochrane Collaboration's risk of bias tool Supporting Table 5:Sensitivity analysis of the correlation between irisin level and global cognition [file BRB3-15-e70662-s001.docx]

| **Databases** | **Search Terms** | **Numbers**  **of Records** |
| --- | --- | --- |
| **Pubmed** | Search: (irisin[Title/Abstract] OR FNDC5[Title/Abstract] OR "Fibronectin Type III Domain-containing Protein 5"[Title/Abstract]) AND (((((((((cogniti*[Title/Abstract]) OR (information processing[Title/Abstract])) OR (attention[Title/Abstract])) OR (intelligence[Title/Abstract])) OR (executive function[Title/Abstract])) OR (memory[Title/Abstract])) OR (dement*[Title/Abstract]))) OR (((("Cognition"[Mesh]) OR "Cognition Disorders"[Mesh]) OR "Cognitive Dysfunction"[Mesh]) OR "Dementia"[Mesh])) | 236 |
| **Cochrane Library** | #1 MeSH descriptor:[Cognition] explode all trees  #2 MeSH descriptor:[Cognition Disorders] explode all trees  #3 MeSH descriptor:[Dementia] explode all trees  #4 #1 OR #2 OR #3  #5 (cogniti* OR "information processing" OR attention OR intelligence OR "executive function" OR memory OR dement*):ti,ab,kw  #6 #4 OR #5  #7 (irisin or FNDC5 or “fibronectin type III domain containing protein 5”):ti,ab,kw  #8 #6 AND #7 | 29 |
| **EMBASE** | #1 irisin:ab,ti OR fndc5:ab,ti OR 'fibronectin type iii domain-containing protein 5':ab,ti  #2 'cognition'/exp OR 'cognitive defect'/exp OR 'dementia'/exp OR cognition:ab,ti OR cognitional:ab,ti OR cognitive:ab,ti OR 'information processing':ab,ti OR attention:ab,ti OR intelligence:ab,ti OR 'executive function':ab,ti OR memory:ab,ti OR dementia:ab,ti OR demential:ab,ti OR dementias:ab,ti  #3 #1 AND #2 | 312 |
| **Web of Science** | (TS=( cogniti* OR "information processing" OR attention OR intelligence OR "executive function" OR memory OR dement*) AND TS=(irisin or FNDC5 or "fibronectin type III domain-containing protein 5") | 322 |
| **Scopus** | TITLE-ABS-KEY ( cogniti* OR "information processing" OR attention OR intelligence OR "executive function" OR memory OR dement* ) AND TITLE-ABS-KEY( "irisin"  OR  "fndc5"  OR  "fibronectin type III domain-containing protein 5" ) | 353 |

**Supplementary Materials**

**Table S1.** Detailed Search Strategy

**Table S2.** Quality assessment of cohort study with NOS scores

| **Item****/Study** | | **Lan 2024** |
| --- | --- | --- |
| Selection | 1.Representativeness of the exposed cohort | * |
|  | 2.Selection of the non exposed cohort | * |
|  | 3.Assessment of exposure | * |
|  | 4.Demonstration that outcome of interest was not present at start |  |
| Comparability | Control for important factor and additional factor | ** |
| Outcome | 1. Outcome assessment | * |
|  | 2.Follow up long enough for outcome to occur | * |
|  | 3.Adequacy of follow up | * |
| Total score |  | 8 |

**Table S3.** Quality assessment of Cross-sectional study with AHRQ score

| **Evaluation Item/Study** | **Kaloğlu 2023** | | |
| --- | --- | --- | --- |
|  | Yes | No | Unclear |
| 1. Define the source of information (survey, record review). | * |  |  |
| 2. List inclusion and exclusion criteria for exposed and unexposed subjects (cases and controls) or refer to previous publications. | * |  |  |
| 3. Indicate time period used for identifying patients. | * |  |  |
| 4. Indicate whether or not subjects were consecutive if not population-based. |  |  | * |
| 5. Indicate if evaluators of subjective components of study were masked to other aspects of the participants. |  |  | * |
| 6. Describe any assessments undertaken for quality assurance purposes (e.g.,test/retest of primary outcome measurements). |  | * |  |
| 7. Explain any patient exclusions from analysis. | * |  |  |
| 8. Describe how confounding was assessed and/or controlled. |  |  | * |
| 9. If applicable, explain how missing data were handled in the analysis. | * |  |  |
| 10. Summarize patient response rates and completeness of data collection. |  |  | * |
| 11. Clarify what follow-up, if any, was expected and the percentage of patients for which incomplete data or follow-up was obtained. | * |  |  |
| Total score | 6 |  |  |

**Table S4.** Quality assessment of randomized controlled trial with the Cochrane Collaboration’s risk of bias tool

| **Evaluation Item/Study** | **Kuster 2017** | | |
| --- | --- | --- | --- |
|  | Low risk of bias | Unclear risk of bias | High risk of bias |
| Random sequence generation (selection bias) | * |  |  |
| Allocation concealment (selection bias) | * |  |  |
| Blinding of participants and personnel (performance bias) |  |  | * |
| Blinding of outcome assessment (detection bias) | * |  |  |
| Incomplete outcome data (attrition bias) | * |  |  |
| Selective reporting (reporting bias) |  | * |  |
| Other sources of bias (other bias) |  | * |  |

**Table S5.** Sensitivity analysis of the correlation between irisin level and global cognition

| **Study of Omission** | **Heterogeneity** | | **Meta-analysis** | |
| --- | --- | --- | --- | --- |
|  | **I^2^** | **p value** | **r（95%CI）** | **p value** |
| Belviranli 2016 | 82% | ＜0.001 | 0.24 (0.07, 0.40) | 0.007 |
| Dicarlo 2024 | 83% | ＜0.001 | 0.27 (0.09, 0.45) | 0.004 |
| Faienza 2021 | 82% | ＜0.001 | 0.25 (0.07, 0.42) | 0.007 |
| Gonçalves 2022 | 82% | ＜0.001 | 0.24 (0.07, 0.41) | 0.006 |
| Ipekten 2024 | 81% | ＜0.001 | 0.29 (0.13, 0.44) | ＜0.001 |
| Kuster 2017 | 83% | ＜0.001 | 0.24 (0.07, 0.41) | 0.006 |
| Li 2024 | 80% | ＜0.001 | 0.24 (0.06, 0.39) | 0.008 |
| Lin 2019 | 50% | 0.04 | 0.32 (0.21, 0.41) | ＜0.001 |
| Lourenco 2020 | 83% | ＜0.001 | 0.25 (0.08, 0.41) | 0.004 |
| Shi 2024 | 83% | ＜0.001 | 0.25 (0.07, 0.42) | 0.007 |
| Zhang2021 | 82% | ＜0.001 | 0.25 (0.07, 0.42) | 0.007 |
| All studies without omission | 81% | ＜0.001 | 0.26 (0.10, 0.41) | 0.002 |
